# The private healthcare market and the sustainability of an innovative community nurses programme based on social entrepreneurship - CoNSENSo project

**DOI:** 10.1186/s12913-018-3513-z

**Published:** 2018-09-05

**Authors:** Ippoliti Roberto, Falavigna Greta, Montani Floriana, Rizzi Silvia

**Affiliations:** 1 Direzione Sanità – Regione Piemonte, Torino, Italy; 2 Istituto di ricerca sulla crescita economica sostenibile (IRCrES) – Consiglio Nazionale delle Ricerche (CNR), Moncalieri, Italy

**Keywords:** Falls, Community nurses, Mountain areas

## Abstract

**Background:**

CoNSENSo is a project funded by the European Union, which is aimed at developing an innovative care model based on community nurses to support active ageing in mountain areas. The planned sustainability of this innovative approach relies on social entrepreneurship on the healthcare market, and this work highlights the necessary conditions for the successful implementation of these entrepreneurial initiatives.

**Methods:**

Considering municipalities in the Piedmont Region and those aged 65 or older as target population, the authors propose several negative binomial regression models to estimate the effectiveness of current private healthcare services in supporting the active aging process. Such effectiveness may represent the ex-ante (positive) reputation of these new social entrepreneurial initiatives on the market.

**Results:**

According to our results, the private supply of healthcare services can effectively support the aging process. Indeed, given that the other predictor variables in the model are held constant, there are statistically significant negative relations between the number of hip fractures and the private supply of healthcare services by dental practitioners and psychologists (*p*-value < 0.05), as well as the private supply of opportunities for social interaction by coffee bars (*p*-value < 0.05).

**Conclusions:**

The authors expect a favourable environment for the entrepreneurial initiatives of community nurses in mountain areas. Accordingly, policy makers cannot reject the hypothesis that the goals reached by the CoNSENSo project may be maintained for the sake of the future generations, avoiding its collapse as soon as public funding shifts to new programmes.

## Background

The COmmunity Nurse Supporting Elderly iN a changing SOciety (CoNSENSo) is an European Union funded project to face one of the most significant European current issue, i.e., the ageing process [1]. Led by the Italian Piedmont Region, the CoNSENSo project brings together 10 partners from Austria, France, Italy and Slovenia and 7 observers representing governmental ministries, health authorities and professional associations from the Alpine Space area. The project expects to create the right conditions to improve health and life quality of senior citizens in the Alpine Space enabling them to stay at home as long as possible, designing and implementing new public policies for elderly care, building adaptable and transferable training modules for nurses and stimulating social entrepreneurship. CoNSENSo project runs for 36 months starting in December 2015, testing the new care model on more than 4000 elderly in 5 pilot study regions: Piedmont and Liguria (Italy), Département du Var (France), Carinthia (Austria) and Slovenia.

In line with the definition of the World Health Organisation (WHO), active and healthy ageing refers to the process of optimizing opportunities for health to enhance quality of life as people age [2]. The project aims at supporting the daily activities of elderly (i.e., those aged 65 or older) through a pro-active approach by Community Nurses (CNs). More precisely, these CNs can successfully organise appropriate interventions to support an active and healthy ageing and to guarantee the supply of necessary healthcare services. The proposed approach can be even more relevant if we consider the limits and difficulties in the utilization of healthcare services among the rural population [3–5], like in this specific case study (i.e., mountain areas).

Current literature highlights that we can support an active and healthy ageing with services aimed at preventing falls [6–9], reducing behavioural and/or environmental risk factors [10–15]. Considering the former type of risk factors, coherently with the current literature, CNs can propose walking groups (i.e., interventions aimed at changing the lifestyle of the elderly) [10–14]. At the same time, considering the latter type of risk factors, CNs can highlight the necessity of adapting the home to reduce hazard risks such as, for example, through the installation of bathroom grab rails (i.e., interventions aimed at promoting environmental modifications) [15]. Obviously, reducing the falls and the related negative outcomes (e.g., hip fracture), we can support the active and aging process in the mountain areas, as well as the sustainability of a healthcare programme relying on CNs [16].

Focusing on the Italian healthcare market, which is the specific case study proposed by the authors, we can identify both public and private providers of healthcare services [17–20]. On the one hand, there are public services currently offered by Local Health Authorities (LHAs) through Family Doctors (FDs), who are responsible for the comprehensive healthcare of patients. Elderly can receive free clinic visits from the FD and, according to the results, specialist exams prescriptions, which will be supplied directly by the LHAs or purchased by them on the healthcare market from public or private providers [17]. On the other one, there are private services supplied by psychologists, physiotherapists, and dental practitioners, which might be purchased directly by the elderly on the healthcare market to support their active aging. This is the scenario in which our innovative programme aims at integrating the current supply of healthcare services. According to the planned new scenario, CNs aim at supporting and at facilitating the daily activities of the elderly and their families directly at their homes, integrating the supply of private healthcare services on the market. For example, current physiotherapists activity on the market is mainly oriented to trauma rehabilitation of young people (ex-post approach); while CNs activity is exclusively oriented to trauma prevention of elderly (ex-ante approach). Note that, the expected sustainability of this innovative approach relies on social entrepreneurship, that is to say, the entrepreneurial initiative of nurses on the healthcare market. In other words, according to the CoNSENSo project, CNs will be private providers on the market, offering their pro-active approach to the elderly and/or other stakeholders (e.g., municipalities, LHAs, relatives and/or families), who may be interested in purchasing these specific services [16]. The proposed approach is coherent with the current age of austerity. Indeed, spending review drives policy makers to rationalize the available resources [21–23] pushing, in some cases, the public healthcare systems to outsource services to the private sector and/or fully privatise them in order to contain costs [24–27]. Obviously, increasing the competitiveness on the healthcare market, we have the possibility to select our healthcare providers [28] and, in this way, to push the market toward the efficient allocation of resources [29, 30], as confirmed in recent analyses [31].

However, the services offered by CNs would be completely new and these entrepreneurs need to leverage the reputation of other private healthcare services to support their business. In other words, in the absence of specific previous experiences, the expected quality of an innovative private healthcare service proposed to the elderly on the market can be supported by other private suppliers. According to this hypothesis, the quality of these services can influence the acceptance of CNs by the elderly and, consequently, the sustainability of the project. More precisely, considering the current period of reduced spending, the authors assume that people will purchase private CN services on the market if, and only if, they have positive expectations about the proposed interventions and the selected supplier (i.e., private or public). Therefore, the precondition for the existence of a social healthcare market in mountain areas is the reputation of the private suppliers that are already on the market, offering similar pro-active approaches able to support active aging [32, 33]. On the one hand, CNs can offer the elderly innovative healthcare and social services aimed at reducing behavioural and/or environmental risk factors. On the other hand, in the absence of previous specific experiences, the elderly will purchase the proposed interventions estimating an expected level of reduction in falls based on their previous experience with other private suppliers on that market. Obviously, as long as the reputation of such private suppliers is positive, without negative experiences or public scandals [34], there will be opportunities for accredited private CNs – and their reputation will be strengthened through successful interventions aimed at changing the lifestyle and/or home environment of the elderly, reducing the occurrence of falls.

These are exactly the assumptions and the hypotheses of our analysis, adopted by the authors to test whether the achievements of the CoNSENSo project could be maintained for the sake of the next generation, avoiding its collapse as soon as European funding shifts to new programmes. More precisely, this work aims to analyse the effectiveness of the current private supply of healthcare services in order to understand whether the environment is favourable for a new service offered by private social entrepreneurs.

## Method

Focusing on the Piedmont Region (i.e., the lead partner of the CoNSENSo project), the authors implemented an empirical analysis to estimate whether the current private supply of health and social services can effectively support the active aging process. We tested the ex-ante reputation of an innovative private service based on the idea of CNs offering their entrepreneurial social initiatives on the healthcare market. In other words, we tried to determine whether favourable conditions exist for this new social business.

The first step in this empirical analysis consisted in selecting the municipalities and target population (i.e., those aged 65 or older), that is to say, the elderly population residing in mountain areas. Then, considering these municipalities and the elements that might support an age-friendly community [35, 36], which represent the proposed observations of the empirical analysis, data were collected about the target population in 2015 (i.e., the most recent available data). In particular, we considered the following:

- number of falls with hip fracture, estimated considering the average number of falls having the selected bad outcome between 2013 and 2015 (data source: Regional Epidemiological Service, Piedmont Region);
- altitude of the town, i.e., the altitude of the main settlement in these municipalities (data source: Italian National Institute of Statistics);
- number of elderly residents, i.e., those aged 65 or older residing in the selected municipality (data source: Italian National Institute of Statistics);
- density of elderly residents, representing a measure of elderly dispersion and equal to the number of elderly residents per square kilometre (data source: Italian National Institute of Statistics);
- total contribution on tax income, representing the public resources available to the municipalities to implement social interventions and equal to a variable percentage of the people income fixed by those municipalities (data source: Ministry of Economy and Finance);
- number of coffee bars, which may represent the private supply of opportunities for social interaction, that is to say, opportunities for the elderly to meet other people in a coffee bar and feel socially included (data source: Chamber of Commerce);
- number of private physiotherapists, representing the private supply of this first specific type of healthcare service to support active aging (data source: Chamber of Commerce);
- number of private psychologists, representing the private supply of this second specific type of healthcare service to support active aging (data source: Chamber of Commerce);
- number of private dental practitioners, representing the private supply of this third specific type of healthcare service to support active aging (data source: Chamber of Commerce);
- percentage of divorced individuals among the elderly residents, representing their first type of marital status (data source: Italian National Institute of Statistics);
- percentage of widowed individuals among the elderly residents, representing their second type of marital status (data source: Italian National Institute of Statistics);
- percentage of married individuals among the elderly residents, representing their the third type of marital status (data source: Italian National Institute of Statistics);
- percentage of unmarried individuals among the elderly residents, representing their last (and residual) type of marital status (data source: Italian National Institute of Statistics).

Using the number of falls with hip fracture as our dependent (count) variable and all the other variables as our independent ones, we tried to determine the most appropriate regression model (i.e., the negative binomial regression model or the Poisson regression model), testing data over dispersion with the likelihood-ratio test of alpha. Once the most appropriate approach was selected, we proposed several models, gradually adding the independent variables. Finally, in order to collect more robust results, we introduced a robust standard error into the regression models.

## Results

**Fig. 1:**
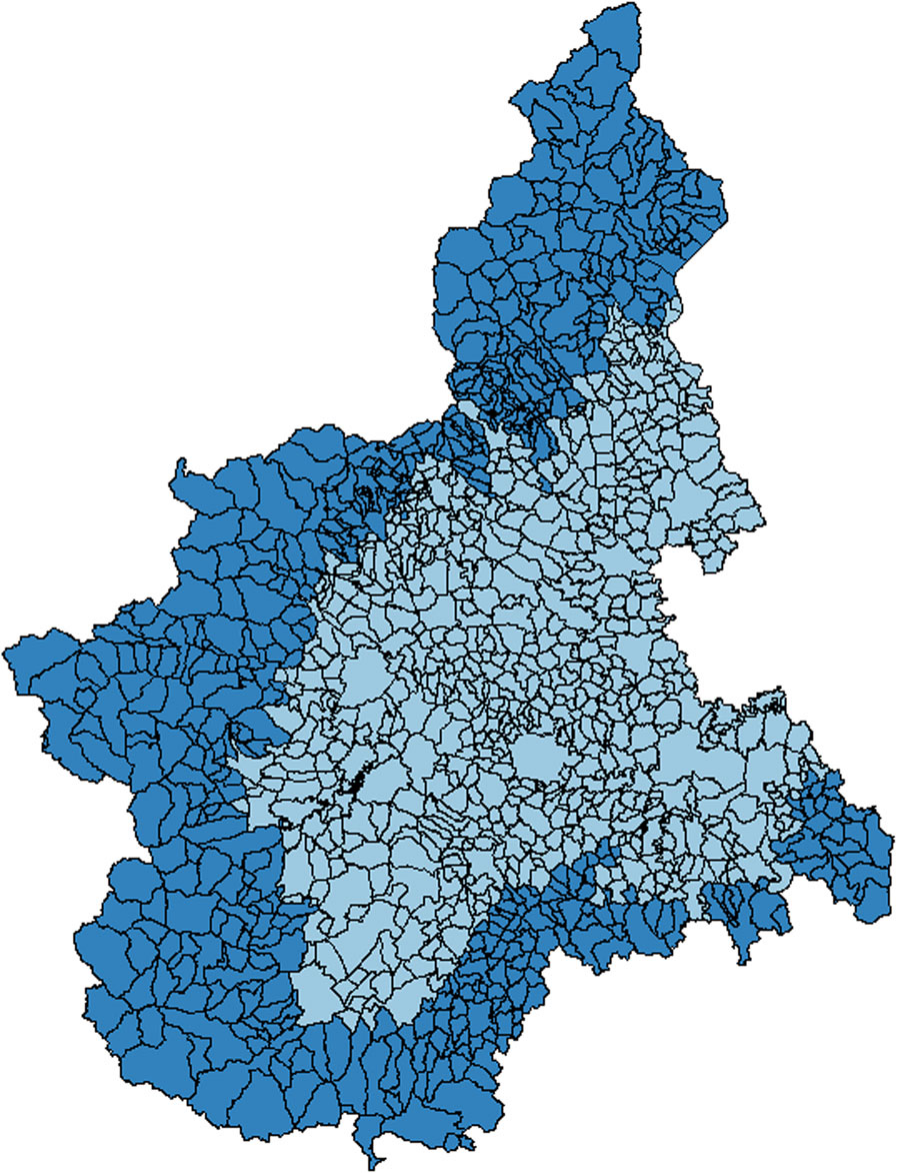
Map of the mountain and plain areas of Piedmont. Mountain areas. Plain areas

The regional public healthcare system in Piedmont is shaped around 13 LHAs, with a variable number of districts and elderly individuals. These LHAs are responsible for the whole elderly population, both in mountain and plain areas, equal to 191,977 and 899,434 individuals respectively in 2015. Figure 1 maps the mountain areas of Piedmont (own elaboration with STATA 12), which were used to select the target population of this innovative care model, while Table 1 proposes some detailed descriptive statistics of Piedmont’s LHAs and their competences, including the number of elderly individuals and municipalities. According to the proposed approach, the municipalities located in mountain areas are the observations of our empirical analysis to test the suggested hypothesis. Table 2 shows some descriptive statistics for these 524 observations, considering the dependent variable (i.e., number of falls with hip fracture) as well as the independent ones (i.e., altitude of the town, number of elderly residents, density of elderly residents, total contribution on tax income, private supply of social and healthcare services, and marital status of the elderly residents).

**Table 1:** Descriptive statistics about LHAs, municipalities and elderly people (65 or older) in 2015

Local Health Authorities	Number of districts	Mountain areas		Plain areas	
		Elderly population	Municipalities	Elderly population	Municipalities
AL	7	9285	49	110,574	146
AT	3	2040	14	50,429	92
BI	2	22,089	50	25,094	21
CN1	6	43,211	127	55,586	48
CN2	2	2894	25	36,929	51
NO	4	732	2	78,583	75
TO1 - TO2	1	–	0	226,012	1
TO3	9	46,230	78	95,882	31
TO4	6	20,255	67	103,827	110
TO5	4	–	0	71,869	40
VC	3	11,795	37	34,213	55
VCO	3	33,446	75	10,436	8
Total	50	191,977	524	899,434	678

Data source: Italian National Institute of Statistics and Piedmont Region

**Table 2:** Detailed descriptive statistics of dependent and independent variables adopted in the empirical analysis

Variables	Obs.	Mean	Std. Dev.	Min	Max
Number of falls with hip fracture	524	2.954	4.756	0.000	36.000
Altitude of the town	524	626.687	294.789	184.000	2035.000
Number of elderly residents	524	366.368	568.381	5.000	4772.000
Density of elderly residents	524	23.276	35.127	0.287	285.089
Total contribution on tax income	524	105,676.900	214,624.300	0.000	1,663,684.000
Number of coffee bars	524	2.926	5.909	0.000	70.000
Number of private physiotherapists	524	1.187	2.846	0.000	31.000
Number of private psychologists	524	0.368	1.122	0.000	10.000
Number of private dental practitioners	524	0.740	2.312	0.000	29.000
Percentage of divorced elderly residents	524	0.026	0.019	0.000	0.143
Percentage of widowed elderly residents	524	0.308	0.065	0.000	0.800
Percentage of married elderly residents	524	0.564	0.078	0.200	0.792
Percentage of unmarried elderly residents	524	0.102	0.051	0.000	0.375

Data source: Italian National Institute of Statistics, Ministry of Economy and Finance, Chamber of Commerce and Piedmont Region

We adopted a Negative Binomial (NB) regression model, after testing data over dispersion with the likelihood-ratio test of alpha (chibar2 equal to 96.53, with *p*-value equal to 0.000). The results of the implemented NB regression models, adopting robust standard errors, are proposed in Table 3. In detail, four models are proposed (A, B, C and D), introducing the independent variables group by group: target population and geographical features (model D), private supply of social services and public resources (model C), private supply of healthcare services to support active aging (model B), and marital status of the elderly (model A). All the models are statistically significant (Wald chi2 test), with an estimated pseudo R-square between 21 and 24%. All the variables are also statistically significant (i.e., 0.1 < *p*-value < 0.01), except the number of private physiotherapists (i.e., *p*-value > 0.1). Note that, since this is a NB regression model, we can interpret the coefficient as follows: for a one unit change in the predictor variable, the difference in the logs of expected counts of the response variable is expected to change by the respective regression coefficient, given the other predictor variables in the model are held constant.

**Table 3:** NB regression model, robust standard error Municipalities located in mountain areas, Piedmont (2015)

Variables	Number of falls with hip fracture			
	Model A	Model B	Model C	Model D
Altitude of the town	−0.000662***	−0.000714***	−0.000708***	− 0.000826***
	(0.000150)	(0.000153)	(0.000154)	(0.000145)
Number of elderly residents	0.00217***	0.00223***	0.00214***	0.00112***
	(0.000281)	(0.000286)	(0.000273)	(0.000122)
Density of elderly residents	0.00238***	0.00292***	0.00287***	0.00298***
	(0.000924)	(0.00100)	(0.000819)	(0.000726)
Total contribution on tax income	−9.35e-07*	−1.01e-06**	−1.51e-06***	
	(4.92e-07)	(5.10e-07)	(4.86e-07)	
Number of coffee bars	−0.0325**	−0.0347**	−0.0534***	
	(0.0145)	(0.0145)	(0.0185)	
Number of private physiotherapists	−0.00540	−0.00245		
	(0.0205)	(0.0215)		
Number of private psychologists	−0.109**	− 0.104**		
	(0.0506)	(0.0525)		
Number of private dental practitioners	−0.0776**	−0.0807**		
	(0.0351)	(0.0346)		
Percentage of divorced elderly residents	4.331*			
	(2.344)			
Percentage of widowed elderly residents	1.641*			
	(0.933)			
Percentage of married elderly residents	2.043**			
	(0.803)			
Constant	−1.274*	0.512***	0.557***	0.696***
	(0.740)	(0.117)	(0.118)	(0.114)
Wald chi2	499.70	452.97	410.12	324.22
Prob > chi2	0.0000	0.0000	0.0000	0.0000
Observations	524	524	524	524

Robust standard errors in parentheses

*** *p* < 0.01, ** *p* < 0.05, * *p* < 0.1

## Discussion

Assuming that the supply of effective private healthcare and social services to support active aging can enhance the ex-ante reputation of a new similar service offered privately, the collected results seem to suggest the existence of a favourable environment for the entrepreneurial initiatives of CNs. Therefore, we cannot reject the hypothesis that the achievements of the CoNSENSo project should be maintained for the sake of the next generation, avoiding its collapse as soon as European funding shifts to new programmes. Indeed, considering the private supply of healthcare services by dental practitioners and psychologists, which can support active aging, there are statistically significant negative relations between their numbers and the number of hip fractures (*p*-value < 0.05). There is no statistically significant relation for what concerns the supply of services by physiotherapists (*p*-value > 0.1), although the coefficient is negative. A potential explanation, which is coherent with the market structure and the current supply of private healthcare services, is the following: physiotherapists activity on the market is mainly oriented to trauma rehabilitation of young people (ex-post approach). CNs activity is exclusively oriented to trauma prevention of elderly (ex-ante approach), representing an integration of this supply of private healthcare services.

Therefore, we cannot reject the hypothesis that a new private intervention by CNs operating as social entrepreneurs may be accepted by society, since the services supplied by private entrepreneurs on the market already enjoy a good reputation. This innovative private intervention could successfully support the active aging of the elderly individuals analysed, despite representing an additional cost for them. Obviously, as confirmed by our results and coherently with economic principles, an increase in the number of suppliers boosts competitiveness on the market, lowering the price of the services and, consequently, maximizing the number of elderly individuals treated. What about the other explanatory variables?

Our results confirm a statistically significant negative relation between the altitude of the municipalities and the number of hip fractures (*p*-value < 0.01), supporting the hypothesis of a higher level of life satisfaction among the elderly depending on the “physical environment”, which can stimulate their active aging [36]. Given that the other predictor variables in the model are held constant (e.g., density and elderly population in model D), the higher the altitude of the town in mountain areas, the lower the expected frequency of falls with hip fracture. This means that valley areas are characterised by a higher occurrence of falls with bad outcomes.

As for social aspects, our results show another interesting relation, which is coherent with the idea of an age-friendly community [35, 36]. The greater the opportunities to spend time together (i.e., cultivate social relations), the lower the number of hip fractures, which is an indicator of successful active aging. The coefficient of available public resources is negative and statistically significant (*p*-value < 0.01 in model C), suggesting that the implementation of social public interventions can enhance active aging, consequently reducing the number of hip fractures. At the same time, the number of coffee bars is related to the private supply of social relations, with a statistically significant impact on reducing falls with bad outcomes (*p*-value < 0.01 in model C). Note that, if the number of predictors increases, the results are still significant, which confirms the robustness of our analysis.

Finally, looking at the marital status of the elderly population investigated, the municipalities with a higher share of divorced elderly individuals are characterised by a higher number of falls with hip fracture, when the other predictor variables in the model are held constant. Indeed, a 1% increase in the number of divorced elderly individuals corresponds to 4 falls with hip fracture, while in the case of married and widowed individuals the expected falls are equal to 2. Note that the dropped category against which the model is assessed is the percentage of unmarried individuals (estimated within the constant). This information represents the available data about the elderly and the composition of their family, suggesting the category at the highest risk of fall and hip fracture.

## Limits

The main limit of this work concerns the availability of information about the elderly population studied, such as, for example, their financial and health conditions. This has forced the authors to make two main assumptions. The first assumption is that there are no significant differences across the municipalities in terms of the health status of the elderly. Moreover, we assumed that the elderly have a minimum income (e.g., pension), so that access to the private healthcare market is not precluded to them. Obviously, this is an oversimplification of the real situation due to data availability, which could partially bias the collected results. Other key factors that can be taken into account to improve the current analysis are the presence of formal and informal caregivers, the health literacy of the elderly, as well as the total number of falls (with or without hip fracture) and the frequency of patient visits from each type of healthcare service provider (e.g., the aforementioned physiotherapists activity).

## Conclusions

People are rational and, from an economic perspective, it means they will use their available resources to purchase goods and services on the market if, and only if, there are positive expectations about the commodities and the suppliers. Taking our specific case study into account, a social healthcare market in mountain areas will exist if, and only if, there are positive expectations about the effectiveness of CN interventions and the reputation of private suppliers already operating on the market, offering similar pro-active approaches able to support the active aging process. In other words, the elderly will purchase the proposed interventions (i.e., services aimed at reducing behavioural and/or environmental risk factors) based both on expected reductions in falls and on their previous experience with similar services. Obviously, as long as there are high expectations about the effectiveness of these services and private suppliers enjoy a good reputation, there will be opportunities for this social business on the market, that is to say, there will exist a demand of CNs services. These are the key theoretical points of our analysis and, according to the results of our empirical analysis, we cannot reject positive expectations about the sustainability of the CoNSENSo project after the conclusion of financial support by European funds.

Obviously, there are other interventions to support an active and healthy ageing such as, for example, medication reviews, medication management modifications, care coordination, addressing psychosocial needs and reviewing goals of care, as well as supply evidence-based outcomes to support claims. The idea of supporting an active and healthy ageing through a pro-active approach (i.e., reducing behavioral and/or environmental risk factors) is a clear simplification of the real world, even if it can be considered a clear “sales pitch” for CNs on the market. Future explorations of the matter should focus on these alternative interventions.

Finally, there are some policy implications. Indeed, both policy makers and public managers of the local healthcare system (i.e., LHAs) may be interested in these innovative services, even though from a different perspective. On the one hand, LHAs may outsource the activities in question to social entrepreneurs, purchasing their services for the elderly population to avoid future costs such as, for instance, the cost of caring for elderly individuals with hip fractures [16]. On the other one, policy makers should involve and coordinate all relevant stakeholders, considering the context in which the innovative approach is introduced, especially when there is no positive ex-ante reputation. In this case, an appropriate sustainability framework would be needed to create a new market for social entrepreneurs in mountain areas, coordinating efforts and social interactions among all relevant stakeholders [37–40].

## Abbreviations

CNs: Community Nurses
CoNSENSo: COmmunity Nurse Supporting Elderly iN a changing SOciety
FDs: Family Doctors
INTERREG: a programme to stimulate cooperation between regions in the European Union funded by the European Regional Development Fund
LHAs: Local Health Authorities
NB: Negative Binomial
