# Correction to: The private healthcare market and the sustainability of an innovative community nurses programme based on social entrepreneurship - CoNSENSo project

**DOI:** 10.1186/s12913-018-3597-5

**Published:** 2018-10-11

**Authors:** Roberto Ippoliti, Greta Falavigna, Floriana Montani, Silvia Rizzi

**Affiliations:** 1 Direzione Sanità – Regione Piemonte, Torino, Italy; 2 Istituto di ricerca sulla crescita economica sostenibile (IRCrES) – Consiglio Nazionale delle Ricerche (CNR), Moncalieri, Italy

## Correction

Following publication of the original article [1], the author reported that their first names and last names were swapped. The original article has been corrected.

The names appeared on the article as follows:

Ippoliti Roberto

Falavigna Greta

Montani Floriana

Rizzi Silvia

They correct names are as follows:

Roberto Ippoliti

Greta Falavigna

Floriana Montani

Silvia Rizzi
